# Results of a worldwide external quality assessment of cfDNA testing in lung Cancer

**DOI:** 10.1186/s12885-022-09849-x

**Published:** 2022-07-12

**Authors:** Jennifer A. Fairley, Melanie H. Cheetham, Simon J. Patton, Etienne Rouleau, Marc Denis, Elisabeth M. C. Dequeker, Ed Schuuring, Kaat van Casteren, Francesca Fenizia, Nicola Normanno, Zandra C. Deans

**Affiliations:** 1 GenQA, Nine, Edinburgh Bioquarter, 9 Little France Road, Edinburgh, EH16 4SA UK; 2 EMQN CIC, Unit 4, Enterprise House, Pencroft Way, Manchester Science Park, Manchester, M15 6SE UK; 3 Medical Biology and Pathology Department, Gustave Roussy, Villejuif, France; 4 Department of Biochemistry and INSERM U1232, Centre Hospitalier Universitaire de Nantes, 9 quai Moncousu, F-44093 Nantes Cedex, France; 5 Department of Public Health and Primary Care, Biomedical Quality Assurance Research Unit, KU Leuven, Kapucijnenvoer 35d, 3000 Leuven, Belgium; 6 Department of Pathology and Medical Biology, University Medical Center Groningen, University of Groningen, Groningen, The Netherlands; 7 IQN Path ASBL, Luxembourg, Luxembourg; 8 Istituto Nazionale Tumori-IRCCS-Fondazione G. Pascale, 80131 Napoli, Italy

**Keywords:** Lung cancer, NSCLC, cfDNA, *EGFR* EQA, Mutation testing

## Abstract

**Background:**

Circulating cell free DNA (cfDNA) testing of plasma for *EGFR* somatic variants in lung cancer patients is being widely implemented and with any new service, external quality assessment (EQA) is required to ensure patient safety. An international consortium, International Quality Network for Pathology (IQNPath), has delivered a second round of assessment to measure the accuracy of cfDNA testing for lung cancer and the interpretation of the results.

**Methods:**

A collaboration of five EQA provider organisations, all members of IQNPath, have delivered the assessment during 2018–19 to a total of 264 laboratories from 45 countries. Bespoke plasma reference material containing a range of *EGFR* mutations at varying allelic frequencies were supplied to laboratories for testing and reporting according to routine procedures. The genotyping accuracy and clinical reporting was reviewed against standardised criteria and feedback was provided to participants.

**Results:**

The overall genotyping error rate in the EQA was found to be 11.1%. Low allelic frequency samples were the most challenging and were not detected by some testing methods, resulting in critical genotyping errors. This was reflected in higher false negative rates for samples with variant allele frequencies (VAF) rates less than 1.5% compared to higher frequencies. A sample with two different *EGFR* mutations gave inconsistent detection of both mutations. However, for one sample, where two variants were present at a VAF of less than 1% then both mutations were correctly detected in 145/263 laboratories. Reports often did not address the risk that tumour DNA may have not been tested and limitations of the methodologies provided by participants were insufficient. This was reflected in the average interpretation score for the EQA being 1.49 out of a maximum of 2.

**Conclusions:**

The variability in the standard of genotyping and reporting highlighted the need for EQA and educational guidance in this field to ensure the delivery of high-quality clinical services where testing of cfDNA is the only option for clinical management.

**Supplementary Information:**

The online version contains supplementary material available at 10.1186/s12885-022-09849-x.

## Introduction

The use of molecular testing to aid determining therapy options in the treatment of non-small cell lung cancer (NSCLC) is now recommended for all patients [1, 2]. Use of formalin-fixed paraffin embedded (FFPE) material from biopsies or resected tumours is considered the gold standard for this testing, however up to 30% of patients do not have suitable material available [3]. This can be due to inaccessibility of the tumour for biopsy, the insufficient amount of tissue or insufficient neoplastic cells for molecular testing. Circulating cell free DNA (cfDNA) has therefore become a recognised alternative source of testing material [1, 4].

cfDNA is comprised of short fragments of DNA (160–170 base pairs) [5, 6] found circulating within a patient’s plasma and also harbours circulating tumour DNA (ctDNA). This ctDNA is shed by tumours and although the mechanisms are not fully understood it has been proposed that processes such as apoptosis and necrosis are responsible [7]. Fragmented DNA may also be released from tumour cells through vesicles [8]. Fragments of DNA from normal dividing cells are also found in the patient’s plasma and, therefore, the fraction of ctDNA in the total amount of cfDNA is often less than 1%. As ctDNA is now in clinical use for the detection of somatic variants in tumours, sensitive mutant detection methods are required to detect the variants [4, 8].

External quality assessment (EQA) is part of a laboratory’s quality assurance process and participation in an EQA scheme has been shown to improve the quality of testing [9–13]. In 2017, four EQA providers working under the umbrella organization, the International Quality Network for Pathology (IQN Path) delivered a pilot EQA involving 32 laboratories to determine the feasibility of offering assessment for cfDNA testing for the detection of defined pathogenic variants in cancer patients. The pilot demonstrated that the EQA could be delivered using artificial plasma with spiked-in pathogenic variants albeit using dry ice to deliver the samples to participating laboratories [14]. The pilot EQA examined the accuracy of *EGFR,* KRAS and *NRAS* variant testing and demonstrated a high-test error rate. In particular, it was observed that for *EGFR* testing, the majority of participants did not detect the c.2369C > T p.(Thr790Met) resistance variant in a case where both this variant and an activating deletion in exon 19 of *EGFR* were present each with a variant allelic frequency (vaf) of approximately 1%. This is a common clinical scenario and laboratories would be expected to detect all variants or at least caveat their clinical report with the limitations of the testing performed.

In addition to the high error testing rate, it was observed that there was a variability in the content of the reports submitted by the participating laboratories. Many laboratories over-interpreted wild-type results and failed to include information considered essential for the correct understanding of laboratory reports. Following release of the EQA results to participating laboratories, an international workshop was held to address some of these issues and also to further discuss areas around testing and reporting of results [4]. Both the original pilot and the subsequent workshop provided guidance for laboratories to improve their testing and reporting in this field [4, 14].

Building on the experience of the 2017 pilot EQA, in 2018 five European EQA providers, Associazione Italiana di Oncologia Medica (AIOM), European Molecular Genetics Quality Network (EMQN), European Society of Pathology (ESP), Genomics Quality Assessment (GenQA) and Gen&Tiss, under the auspices of IQNPath, provided an EQA for the testing of *EGFR* variants in cfDNA for NSCLC patients. Participation was open to any interested laboratory and 264 laboratories participated. In this paper we present the results of the 2018 EQA and include countries represented, methodologies employed, and the mutant detection rates determined. We demonstrate that a large-scale assessment can be delivered by multiple EQA providers, against the same standards, to promote high-quality testing and reporting in this field.

## Materials and methods

### EQA design

Five EQA provider members of IQN Path provided the EQA. It was designed to assess laboratories on the testing and reporting of plasma cfDNA for the presence of *EGFR* variants in the context of NSCLC*.* Participants registered through a single EQA provider and each EQA provider was responsible for the distribution of samples to their registered laboratories. Five samples were distributed for testing and all participating laboratories were supplied with identical materials. Participants were requested to test the samples according to their routine testing protocols and submit fully interpretative reports tailored to address the clinical questions detailed in the mock clinical case scenarios provided. Laboratories returned their results to the EQA provider with which they had registered. In addition, participants were requested to complete an online survey to capture relevant information, although this was not compulsory. The EQA was carried out according to the requirements of the International Standard ISO/IEC 17043:2010 [15].

Laboratories were provided with an individual score report and each EQA provider produced a general report for all their participating laboratories. In addition, an overall report of the results from *all* EQA providers was provided. Participating laboratories had the right to appeal if they disagreed with their scores by the assessment procedure. Appeals were considered anonymously by the expert assessment team and if upheld, scores were amended as required.

### Samples and validation

The EQA samples were manufactured to contain 250 ng of cfDNA fragmented to an average fragment size of 170 bp mimic patient cfDNA according to proprietary procedures by Horizon Dx (Cambridge, UK) Each sample was supplied as 3 mL aliquots of artificial plasma spiked with the cfDNA. The genotypes and VAF were determined by the EQA providers and incorporated common sensitising and resistance mutations at a range of frequencies. All were expected to be encountered through clinical service. Table 1 summarises the *EGFR* genotypes of the EQA samples. The samples were validated prior to distribution by five laboratories using a range of commonly used techniques to determine whether the expected genotyping results were obtainable and of reportable quality in different laboratories (Table 2) The techniques used were next generation sequencing (Oncomine™ Lung cfDNA Assay (ThermoFisher) and GeneRead™ QIAact Lung UMI (Qiagen)), real time PCR (.therascreen® EGFR Plasma RGQ PCR Kit (Qiagen) and cobas® EGFR Mutation Test v2 (Roche)) and digital droplet PCR (Biorad and Stilla assays) The samples were distributed to the validating and participating laboratories at ambient temperature and storage at 4 °C upon receipt was recommended.

**Table 1:** Summary of clinical cases and expected results

Sample	Case	Reason for referral	Genotype
IQN Path Sample 2018 – A	1	Never smoker patient, diagnosed with metastatic lung adenocarcinoma at age 62. *EGFR* testing performed on the patient’s tumour biopsy specimen failed. Testing for *EGFR* gene mutations on the patient’s plasma sample has been requested.	c.2236_2250del p.(Glu746_Ala750del) (1.3% VAF)
IQN Path Sample 2018 – B	2	Patient with metastatic lung adenocarcinoma diagnosed at age 80. After resection, tumour tissue was analysed and no *EGFR* variant was detected. *EGFR* gene testing has been requested on the patient’s plasma sample.	No mutations detected within regions tested
IQN Path Sample 2018 – C	3	Patient with metastatic lung adenocarcinoma, diagnosed at age 68. Patient received first line EGFR*-*TKI treatment and is now in clear clinical progression. No tissue sample or cytology specimen of progressing disease is available due to their poor clinical condition. Testing of the patient’s plasma sample for *EGFR* gene variants has been requested.	c.2369C > T p.(Thr790Met) (5.1% VAF) and c.2573 T > G p.(Leu858Arg) (4.7% VAF)
IQN Path Sample 2018 – D	4	Patient diagnosed with metastatic lung adenocarcinoma at age 55. The patient was found to have an *EGFR* mutation and received first line treatment with an EGFR-TKI. At progression of the disease on TKI, the patient had a tissue biopsy but no tumour cells were present. *EGFR* gene testing has been requested on the patient’s plasma sample	c.2236_2250del p.(Glu746_Ala75del) (6.2% VAF)
IQN Path Sample 2018 – E	5	Patient diagnosed with *EGFR*-mutant metastatic lung adenocarcinoma at age 65. The patient has a radiological progression of their primary tumour wheras the metastatic lesions are stable. Testing for *EGFR* gene variants on patient’s plasma sample has been requested.	c.2369C > T p.(Thr790Met) (0.81% VAF) and c.2573 T > G p.(Leu858Arg) (0.49% VAF)

**Table 2:** Validated results for EQA samples

	Sample / Case					Testing method
	A / 1	B / 2	C / 3	D / 4	E / 5	
Expected results (manufacturer’s data using ddPCR)	c.2236_2250del p.(Glu746_Ala750del) (1.3% VAF)	WT	c.2369C > T p.(Thr790Met) (5.1% VAF) and c.2573 T > G p.(Leu858Arg) (4.7% va)	c.2236_2250del p.(Glu746_Ala750del) (6.2% VAF)	c.2369C > T p.(Thr790Met) (0.81% VAF) and c.2573 T > G p.(Leu858Arg) (0.49% VAF)	
Laboratory 1	Deletion in exon 19 (uncharacterised)	WT	c.2369C > T p.(Thr790Met) c.2573 T > G; p.(Leu858Arg)	Deletion in exon 19 (uncharacterised)	c.2573 T > G p.(Leu858Arg)	therascreen® EGFR Plasma RGQ PCR Kit (Qiagen)
	c.2236_2250del p.(Glu746_Ala750del) (1.11% VAF)	WT	c.2369C > T p.(Thr790Met) (4.46% VAF) c.2573 T > G p.(Leu858Arg) (5.10% VAF)	c.2236_2250del p.(Glu746_Ala750del) (6.26% VAF)	c.2369C > T p.(Thr790Met) (0.66% VAF) c.2573 T > G p.(Leu858Arg) (0.33% VAF)	Oncomine™ Lung cfDNA Assay (Life Technologies) on S5XL System (Life Technologies)
	c.2236_2250del p.(Glu746_Ala750del) (1.17% VAF)	WT	c.2369C > T p.(Thr790Met) (4.56% VAF) c.2573 T > G p.(Leu858Arg) (3.72% VAF)	c.2236_2250del p.(Glu746_Ala750del) (2.62% VAF)	c.2573 T > G p.(Leu858Arg) (1.35% VAF)	GeneRead™ QIAact Lung UMI Panel (Qiagen) on GeneReader NGS System (Qiagen)
Laboratory 2	Deletion in exon 19 (uncharacterised)	WT	c.2369C > T p.(Thr790Met) c.2573 T > G p.(Leu858Arg)	Deletion in exon 19 (uncharacterised)	c.2573 T > G; p.(Leu858Arg)	Stilla ddPCR Technologies
Laboratory 3	Deletion in exon 19 (uncharacterised)	WT	c.2369C > T p.(Thr790Met) c.2573 T > G p.(Leu858Arg)	Deletion in exon 19 (uncharacterised)	c.2369C > T p.(Thr790Met)	cobas® EGFR Mutation Test v2 (Roche)
Laboratory 4	N/A	N/A	c.2369C > T p.(Thr790Met) c.2573 T > G p.(Leu858Arg)	N/A	c.2369C > T p.(Thr790Met)	cobas® EGFR Mutation Test v2 (Roche)
	Deletion in exon 19 (uncharacterised) (2.7% VAF)	WT	c.2369C > T p.(Thr790Met) (4.5% VAF) c.2573 T > G p.(Leu858Arg) (5.2% VAF)	Deletion in exon 19 (uncharacterised) (5.6%)	c.2369C > T p.(Thr790Met) (0.65% VAF) c.2573 T > G p.(Leu858Arg) (0.59% VAF)	ddPCR (BioRad-assays)
Laboratory5	N/A	N/A	c.2369C > T p.(Thr790Met) c.2573 T > G p.(Leu858Arg)	N/A	c.2369C > T p.(Thr790Met)	cobas® EGFR Mutation Test v2 (Roche)

*N/A* Not analysed; *WT* wild type (no mutation detected); *ddPCR* digital droplet PCR; *Ex19del* deletion in exon 19 of *EGFR*; *VAF* variant allele frequency

Nomenclature according to *EGFR* gene reference sequence LRG_304t1

### Assessment

Assessment was performed by two expert advisors against peer-assessed marking criteria (Supplementary Table 1). Each EQA provider was responsible for the overall assessment of their registered laboratories. A harmonisation meeting between all the EQA providers ensured that the marking was standardised and any issues across participating laboratories were discussed, resulting in a uniform approach to assessment.

Participant reports were assessed for genotyping accuracy. In addition, four of the EQA providers (EMQN, ESP EQA, Gen&Tiss and GenQA) also reviewed the interpretation of the genotypes provided and the clerical accuracy of the reports. All categories were marked out of a maximum of 2 points; marks were deducted for genotyping errors and where aspects of a report considered to be essential were missing.

The standard of performance was based solely on the genotyping score; laboratories were considered poor performers if they scored 0 for any of the cases i.e., reported an incorrect genotyping result (false positive and/or false negative).

### Computational and statistical analysis

EQA participant and validation data were analysed using Microsoft Excel 2013 (Microsoft, Redmond, WA, United States of America). The overall error rate was calculated by dividing the total number of false positive, false negative and ‘incorrect variant reported’ results over the total number of genotypes reported by the participants. Technical failures and unmarked reports were not included in the total numbers.

## Results

### Participation

**Fig. 1:**
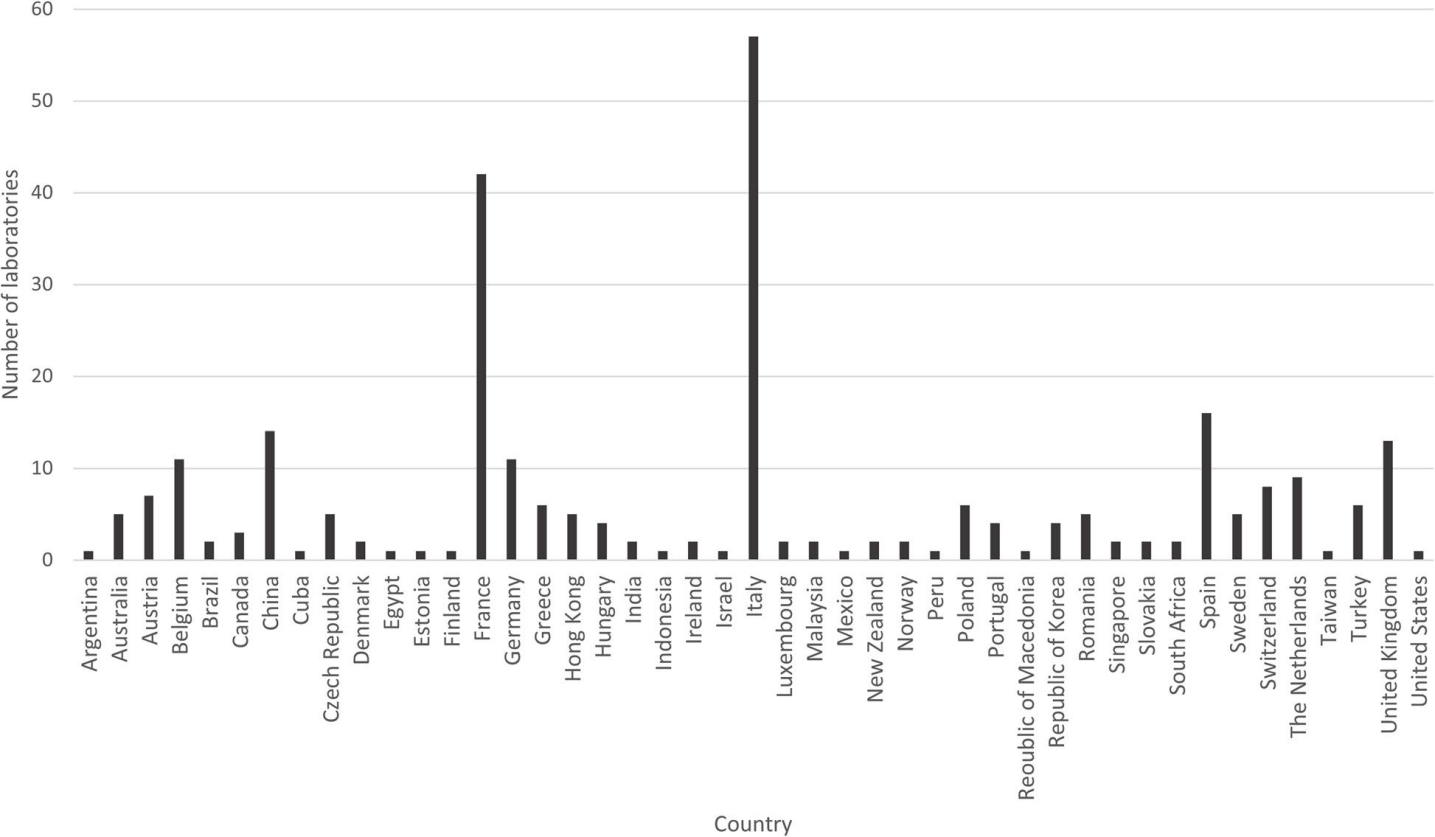
Summary of geographical location of laboratories

A total of 304 laboratories from 45 different countries registered across the five EQA providers (Fig. 1) and 264 (87%) submitted results. Two of the EQA providers are National Schemes (AIOM – Italy and Gen&Tiss - France) therefore the laboratories participating with these providers were from single countries.

Five laboratories were unable to extract cfDNA from the artificial plasma samples provided. All five of these laboratories used the same DNA extraction method which suggested an issue of incompatibility of the artificial plasma with the particular DNA extraction technique (Helix Circulating Nucleic Acid (Diatech Pharmacogenetics, Italy); these laboratories were excluded from the results of the EQA. No other laboratories using this method participated in the EQA.

A total of 1316 reports were assessed. One laboratory only submitted results for case 1, without explanation and was excluded from the results for the other cases. The technical failure rate of the EQA was just under 2% (26/1316 samples reported). A small number of laboratories reported leaking of the plasma from the tubes during transportation; the majority were supplied with replacement samples to ensure they were not disadvantaged by sub-optimal EQA material. An audit was performed of the EQA results from laboratories that did test the leaking samples and all reported the correct genotyping results, confirming that the leakage did not cause any subsequent testing issues.

Three laboratories had a least one of their reports not marked; one laboratory submitted screenshots of the online survey and the data was not assessed for all the cases, one laboratory did not perform testing of cases 1 and 2 as their testing strategy only included testing of recurrence cases for the *EGFR* c.2369C > T p.(Thr790Met) variant; the scenario provided for these cases was for a primary tumour so these cases were not marked for this laboratory and the third laboratory did not submit a report for case 5.

### Genotyping accuracy

The mean scores obtained by participating laboratories are shown in Table 3. The genotyping results were further sub-divided as to the accuracy of the result or type of error made (Table 4).

**Table 3:** Mean scores of all participating laboratories (maximum score = 2)

Category	Case 1	Case 2	Case 3	Case 4	Case 5	All cases
Mean Genotyping Score	1.83	1.93	1.82	1.86	1.83	1.85
Mean Interpretation Score^a^	1.58	1.45	1.54	1.40	1.49	1.49
Mean Clerical Accuracy Score^a^	1.89	1.9	1.9	1.89	1.89	1.89

^a^Not assessed for AIOM laboratories

**Table 4:** Breakdown of results submitted by participating laboratories

	Case 1	Case 2	Case 3	Case 4	Case 5
Correct result and correct nomenclature used	106	248	193	122	115
Correct result (variant uncharacterised)	125	N/A	10	88	4
Correct result (incorrect nomenclature)	12	N/A	43	39	26
One of the two variants present not reported	N/A	N/A	7	N/A	79
False negative result	13	N/A	3	3	24
False positive result	2	5	1	5	0
Incorrect *EGFR* variant reported	1	N/A	1	1	6
Technical failure	3	8	4	4	7
Not marked	2	2	1	1	2

*N/A* Not applicable

Overall, the results for cases 1, 2, 3 and 4 were of a very high standard with most laboratories reporting the correct genotyping results (92% for case 1, 94% for case 2, 94% for case 3 and 95% for case 4) not taking into account the correct use of mutation nomenclature. There were 19 (1.8%) false negative results, 13 (1.2%) false positive results and 3 (0.3%) reports where an incorrect *EGFR* variant was reported. The sample provided for case 3 had two *EGFR* variants present: seven (2.7%) laboratories only reported the presence of one variant.

Cases 1 and 4 both contained the same deletion in exon 19 of the *EGFR* gene. Laboratories used incorrect nomenclature to describe the variants as follows: 12 (5%) of laboratories in case 1 and 39 (15%) of laboratories in case 4, resulting in genotyping score deductions. For case 1, 125 (46%) laboratories and 8 (33%) for case 4 were unable to characterise the mutations due to the technology used for testing (mainly PCR based technologies) which did not enable the mutation present to be fully characterised. These laboratories were not penalised because the exact genotyping is not required for treatment-decision-making. The case 3 variants were reported using incorrect nomenclature by 43 (16%) laboratories resulting genotyping score deductions.

Case 5 was a more challenging sample with two variants present in *EGFR:* c.2369C > T p.(Thr790Met) at 0.81% allelic frequency and c.2573 T > G p.(Leu858Arg) at 0.49% allelic frequency. The variant allele frequencies (vafs) for both had previously been measured using digital droplet PCR by the commercial manufacturer of the materials. Validation results for these samples were inconsistent with regards to the detection of the presence of the *EGFR* c.2573 T > G p.(Leu858Arg) variant and the c.2369C > T variants in the sample (see Table 2). Two of the validation techniques (Oncomine™ Lung cfDNA assay and Bio-Rad ddPCR assay) were able to consistently detect both mutations but the cobas® EGFR Mutation Test v2 (Roche) was not in 3 independent laboratories, along with Silla ddPCR technology, the therascreen EGFR Plasma RGQ PCR kit (Qiagen) and GeneRead™ QIAact Lung UMI Panel (Qiagen) on GeneReader NGS System (Qiagen). In addition, inconsistency in the reporting of the results was observed between laboratories using the same methodology during the EQA. For example of the 72 laboratories using cobas® EGFR Mutation Test v2 (Roche), only 18 (25%) reported the presence of both variants, a further 42 (58%) only reported the presence of the c.2369C > T p.(Thr790Met) variant, one laboratory (1%) reported no variants and one laboratory (1%) reported a false positive. Due to this inconsistency in the validation and during the EQA and the fact that only 55% of laboratories reported the correct result, a problem with the sample could not be ruled out and therefore the marking criteria was adjusted for this case as detailed in Supplementary Table 2. This ensured that the sample was included in the EQA assessment, and that laboratories were only deducted a full 2 marks if they reported an incorrect *EGFR* variant. The marking was also dependent on the sensitivity of the assay used, as reported by laboratories. If a laboratory reported a limit of detection (LOD) of their assay higher than that of the validated variant allele frequency of the specific variant, no marks were deducted.

The case 3 sample had two *EGFR* variants, c.2573 T > G p.(Leu858Arg) and c.2369C > T p.(Thr790Met) with VAF 4.7 and 5.1% and 246 of 263 participants submitting reports for this case (94%) correctly detected both variants. Interestingly, in case 5 with the same variants but now with VAF 0.49 and 0.81%, both variants were reported by only 145 of 263 participants (55%). Twenty-four (9%) laboratories did not report the presence of both *EGFR* variants and 79 (33%) laboratories stated the presence of only one variant: 62 (23%) reported the presence of the c.2369C > T p.(Thr790Met) variant only. In addition, there were six (2%) laboratories that reported an incorrect *EGFR* mutation in this case. Taking into account the LOD of the assays used and described clearly in the report, along with the revised marking criteria, the mean genotyping score for this case (see Supplemental Table 2), however, was concordant to that of the other four cases (Table 3).

### Testing methodologies

**Fig. 2:**
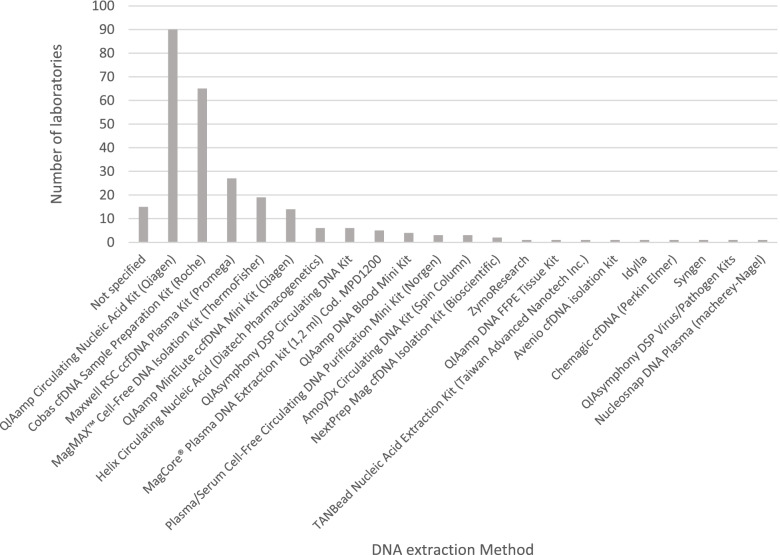
Summary of DNA extraction methods used by participating laboratories

The methods used by laboratories to extract DNA and test the samples were collected using the online survey and the submitted EQA reports. The five most frequently used commercial kits for DNA extraction were used by more than 80% of participating laboratories (see Fig. 2). All were designed specifically for cfDNA extraction. Extraction kits not specifically designed for use for cfDNA were also applied by some laboratories e.g. QIAamp DNA Blood Mini Kit and the QIAamp DNA FFPE Tissue Kit. No specific data regarding the performance of these kits in this EQA is available. As mentioned above, an issue was observed with laboratories using the Helix Circulating Nucleic Acid (Diatech Pharmacogenetics) and it is likely that the artificial plasma used was incompatible with the commercial DNA extraction method.

**Fig. 3:**
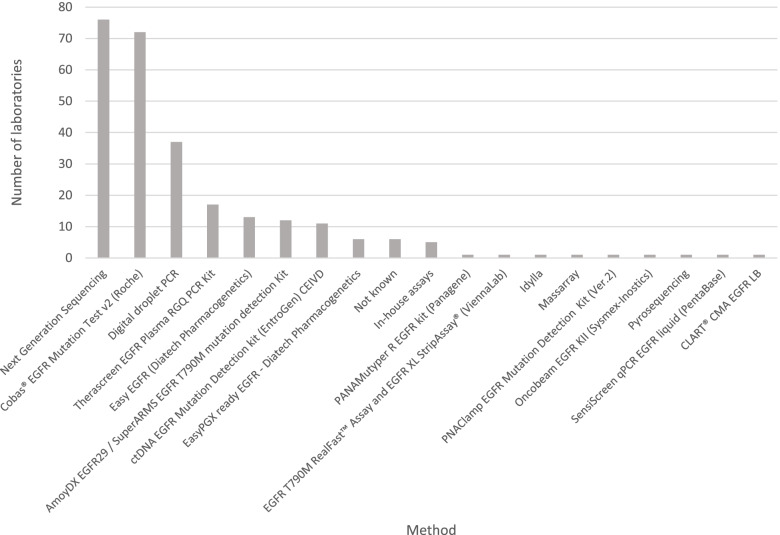
Summary of testing methods used by participating laboratories

**Fig. 4:**
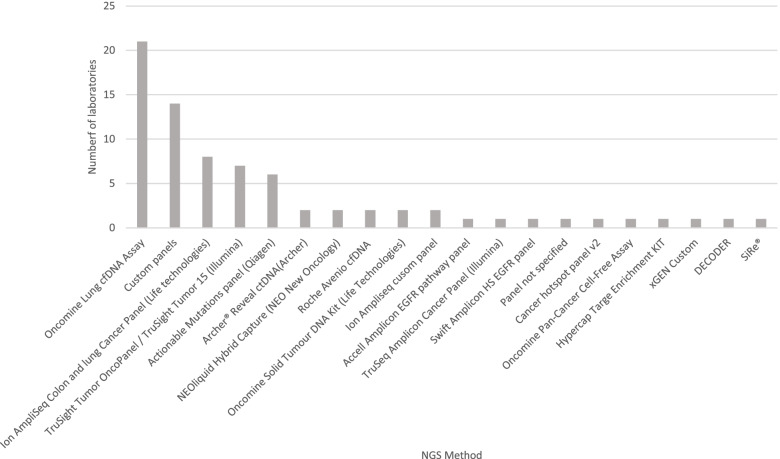
Breakdown of NGS panels used by participating laboratories

The most frequently used testing strategy was Real-Time PCR (51% of laboratories) (Fig. 3) of which the cobas® EGFR Mutation Test v2 (Roche) assay was the most commonly used test (27% of total laboratories). Next Generation Sequencing (NGS) represented the next most common testing strategy used by participating laboratories (27% of laboratories). A breakdown of the NGS panels used is shown in Fig. 4. The most frequently used panel was the Oncomine™ Lung cfDNA Assay (8% of all laboratories). No other NGS panel was used by more than eight laboratories with the majority of panels being used by only one or two participants. As was the case for DNA extraction methods, some laboratories used tests that were not specifically designed for cfDNA, including NGS panels and other technologies.

There was no correlation between any testing method and results obtained for the EQA. As the EQA examined the end-to-end testing strategy then both DNA extraction and testing methods were combined and the performance of individual test methods was not possible.

### Reporting of results

Laboratories registered for this EQA with EMQN, Gen&Tiss, ESP-EQA and GenQA were awarded scores for the interpretation of their reports (Table 3) using the expert opinion on the contents of cfDNA reports from an international workshop [4, 14] as a basis for the marking criteria (Supplementary Table 1). The mean scores for interpretation of the cases were lower than those for genotyping.

The majority of reports (54%) for cases 1, 3 and 4 correctly described the mutations using Human Genome Variation Society (HGVS) nomenclature [16]. A further 28% of reports stated that techniques were used where it was not possible to characterize the variants detected and therefore use of HGVS nomenclature was not required. Some reports (12%) used either incorrect nomenclature or only reported the results at the amino acid level. General omissions from the reports included failure to provide correct gene reference sequences, reporting the sample type incorrectly (e.g. FFPE instead of plasma) or use of the terms ‘positive’ or ‘negative’ to describe the mutation status.

## Discussion

cfDNA testing is now a recognised method for testing for *EGFR* variants in NSCLC to influence targeted therapy treatment decisions [1], with its use complementing that of testing of tumour tissue. There is therefore a requirement to ensure that such testing is accurate and reported in a clear and precise manner. EQA can play an important role in demonstrating this by providing an external measure of the standard of a clinical service and providing educational support when appropriate [17–19].

EQA enables the benchmarking of laboratory services through the provision of the same material to be tested and reported. The use of real patient material for large scale cfDNA EQAs is not feasible due to the amount of patient plasma that would be required. Previous EQAs delivered for testing of cfDNA *EGFR* variants have used alternative materials, for example, [20] provided DNA for testing but this approach had a limitation in that the complete testing process, including DNA extraction, was not able to be assessed. An EQA using plasma or blood samples spiked with DNA [14, 21] were delivered to a relatively small number of participants with sample transportation either temperature controlled by use of dry ice, or by next day delivery, if participants were localised to the same country. Such approaches are feasible when the volume of packages are small and the geographical distance travelled is minmised. In order to deliver EQA to multiple participants worldwide, this EQA utilised artificial plasma which enabled distribution at ambient temperature as it remained stable for a few days, long enough to reach participants in multiple continents. The procurement of the artificial plasma was harmonised between the five European EQA providers which enabled a large-scale comparision of laboratories globally and allowed increased efficiency and reduced cost of the material used. However, the artificial plasma appeared incompatible with one particular commercial DNA extraction method, but the technical failure rates reported were less than 2%, suggesting that the material is suitable for most DNA extraction methods. Today only a few interlaboratory comparisons studies for somatic testing were performed to validate various cfDNA extraction methods with multiple testing methods, mostly involving a small numbers of participants using spiked-in DNA in pooled plasma [22], commercial refence-material [23] and patients-derived plasma [24]. Future EQA runs involving > 250 participants would benefit from more extensive validation by incorporating multiple DNA extraction methods with multiple testing methods.

The overall genotyping error rate was 11.7% which was lower than that reported for previous EQAs where blood or plasma was the initial starting material [14, 21] but slightly higher than that where extracted cfDNA only was distributed [20]. In this EQA the error rate for samples harbouring no pathogenic variants or *EGFR* variants present at an allele frequency of above 1% (cases 1–4) was 4.1% (42/1028). However for the sample which had two variants in *EGFR* present both below 1% (case 5), the error rate was 43% (109/254). It is recognised that the genotype of this sample was challenging and participating laboratories may not have used testing stategies with sufficient sensitivity to detect alleles at such low frequencies. Limited detection of these variants may also be assigned to the use of methods that were not specifically designed for cfDNA, both for extraction and testing. It is expected that this approach will be impacted upon when the IVD R is implmented and laboratories and the diagnostic industry will be required to complete robust validtaions fo rdefined clinical utility [25]. An issue with the EQA sample cannot be excluded as testing gave inconsistent results during validation with only two of the techniques able to detect both the *EGFR* c.2573 T > G p.(Leu858Arg) and the *EGFR* c.2369C > T p.(Thr790Met) present.. Furthermore, in addition to having low variant allele frequencies for both variants, the *EGFR* c.2369C > T p.(Thr790Met) is associated with acquired resistance to EGFR tyrosine kinase inhibitors (EGFR-TKIs) [26] and would be expected to be present at a lower level than the original sensitising mutations in a tumour. However, in case 5 of this EQA the c.2369C > T varriant was present at a higher level that the sensitising c.2583 T > G sensititising variant The sample therefore may not be truly representatvie of a clinical sample and assays may not be optimised for this scenario which may account for some of the variability in genotyping seen. Analysis of the same *EGFR* p.(Leu858Arg) and p.(Thr790Met) mutations with higher VAF (4.7 and 5.1%) in case 3 revealed correct identification of both variants by 246 of 263 participants (94%). This illustrates that the assays used overall have high specificity for these mutations. Although the genotyping case 5 was comparable to the analysis of the other cases taking into account the LOD of the assays used, the results from case 5 demonstrated that the detection of mutations at VAF < 1% requires more sensitive assays than those used by > 40% of the participating laboratories.

The scope of *EGFR* testing was variable. A small number of laboratories performed testing only for the c.2369C > T p.(Thr790Met) resistance variant. In the case of first line treatment with EGFR tyrosine kinase inhibitors (as in cases 1 and 2) it is inappropriate to only test for this resistance variant as this strategy will not provide the correct information for a clinician to determine the possibility of response to treatment. In the case of progressive disease, it is good practice to test for the original *EGFR* variant identified as this can provide useful data that tumour DNA has been tested and also on the sensitivity of the assay performed i.e. if a resistance mutation is not detected and the original variant is not detected, consideration must be given to the likelihood of insufficient test sensitivity [27]. However, it must be acknowledged that this EQA was run in 2018, when osimertinib was available only for patients progressing on EGFR TKI and with a p.(Thr790Met) variant [28]. The increasing use of osimertinib in the first line therapy of *EGFR* mutant lung cancer is leading a change to testing strategies. Although the same actionable *EGFR* mutations (e.g. deletions in exon 19 and c.2573 T > G p.(Leu858Arg) are also targets for osimertinib at first line, the resistance mechanisms on osimertinib are very different and do only show very low occurrence of T790M [28].

Including the scope of testing on the report is also of importance as assays may not be able to detect all clinically relevant variants e.g. insertions of exon 20 in *EGFR* or in the case of ddPCR less common hotspot variants such as those at position 719 or 861 of *EGFR.*

Many laboratories over-interpreted a “no mutations detected” result, advising that the absence of a mutation indicated that the patient would be unlikely to respond to EGFR TKIs. This interpretation is inappropriate, and more caution is advised; cfDNA analyses are known to have reduced sensitivity for detecting variants [29–31] and there is a chance that either no tumour DNA was tested or the variant level was below the limits of detection of the assay performed. As observed in the IQNPath pilot EQA delivered in 2017 [14], some reports provided insufficient information regarding the limitations of the test performed; these are required to determine how reliable a negative result is [4]. This will impact on the clinical interpretation of a test result with a negative result in a less sensitive assay being more likely to be a false negative. Circulating DNA has been shown to represent a small fraction (< 1%) of total cell free DNA [32] therefore detection of variants with allelic frequencies below 1% in cfDNA is thought to be important. As not all techniques have the same limits of detection, including inter-laboratory, it is important for this information to be included in clinical reports so the reader can determine whether there is a likelihood of a false negative result.

To ensure standardization in the reporting of genomic changes, the use of HGVS nomenclature and inclusion of an appropriate reference sequence in reports is important [33]. Laboratories followed HGVS guidance variably. The most frequent error was in the reporting of variants using only the predicted amino acid change(s) and not reporting the nucleotide variant(s). As the assays performed are DNA based tests, the variants detected should be described in terms of the nucleotide change(s) and ideally with the predicted amino acid changes also provided.

## Conclusions

This EQA demonstrated the feasility of delivering a large worldwide EQA assessing accuracy and reporting of cfDNA testing for *EGFR* variants in lung cancer. However, the EQA has highlighted issues across the process in both testing of the samples and reporting of the results. Laboratories should follow up any errors highlighted in this and endeavour to implement process to improve the clinical pathway. Subsequent runs of EQA should aid in the quality assessment process.

## Supplementary Information

**Additional file 1.**

## Data Availability

The datasets generated during and/or analysed during the current study are available from the corresponding author on reasonable request.

## Abbreviations

AIOM: Associazione Italiana di Oncologia Medica
cfDNA: Circulating cell-free DNA
ddPCR: Droplet digital polymerase chain reaction
EGFR: Epidermal growth factor receptor
EMQN: European Molecular Genetics Quality Network
EQA: External quality assessment
ESP: European Society of Pathology
GenQA: Genomics Quality Assessment
HGVS: Human Genome Variation Society
IQNPath: International Quality Network for Pathology
NGS: Next Generation Sequencing
LOD: Limit of detection
NSCLC: Non- small-cell lung cancer
QC: Quality control
VAF: Variant allele frequency
