# Supplementary material for: Results of a worldwide external quality assessment of cfDNA testing in lung Cancer
Source: BMC Cancer. 2022 Jul 12;22:759. doi: 10.1186/s12885-022-09849-x (PMC9275131; doi:10.1186/s12885-022-09849-x)
Supplement: Supplementary file 1 — Additional file 1. [file 12885_2022_9849_MOESM1_ESM.docx]

***Supplementary Table 1*** *– Marking criteria applied to the EQA*

| **Marking Category** | **Criterion** | **Deduction** |
| --- | --- | --- |
| Genotyping | Correct result reported (method enables mutation characterisation AND correct HGVS nomenclature used) | 0 |
|  | Correct result reported (method does not enable mutation characterisation) | 0 |
|  | Correct result reported within the limitations of the test performed | 0 |
|  | False positive result in any gene reported (critical genotyping error) | 2 |
|  | False negative result reported (mutation is present below limit of detection or reporting cut-off of assay) | 0 |
|  | False negative result reported (not known if mutation is present below limit of detection or reporting cut-off of assay: critical genotyping error) | 2 |
|  | Incorrect mutation reported (critical genotyping error) | 2 |
|  | Only 1 mutation reported (for cases with 2 mutations and limit of detection or reporting cut off below variant frequency; critical genotyping error) | 2 |
|  | Mutation described incorrectly e.g. incorrect deletion reported at nucleotide or amino acid level (non-critical genotyping error) | 0.5 |
|  | No (or incorrect) HGVS nomenclature used | 0.5 |
|  | Minor HGVS error | 0 |
|  | Technical failure (see comments) | 1 |
|  | Mutation reported at protein level not nucleic acid level | 0.5 |
|  | Method performed is able to characterise the variant but the characterised result has not been reported | 0.5 |
|  | SNP reported but not identified as SNP | 0.5 |
| Interpretation | All essential interpretative elements provided | 0 |
|  | Critical interpretation error | 2 |
|  | Misleading interpretive comment | 1 |
|  | It is recommended to state that the analysis of a plasma sample is not 100% sensitive and therefore the presence of a mutation may have been missed (refers to cases 2 and 4 only) | 0 |
|  | Over / inappropriate interpretation of a negative (or normal) result using cfDNA. For example, advising that the absence of the mutation indicates that the patient would be unlikely to benefit from an EGFR TKI (refers to cases 2 and 4 only) | 0.5 |
|  | Incorrect sample type reported (e.g. FFPE instead of Plasma) | 0.2 |
|  | No statement about the methodology performed | 0.5 |
|  | Failure to provide any, or insufficient, details of the scope of the test and/or limitations of the test performed, in relation to the suitability of the material provided. | 0.2 |
|  | Insufficient information provided on the NGS testing methodology - platform, and/or manufacturer, and/or strategy (i.e. WES, targeted) not listed on report | 0.2 |
|  | Incorrect and/or inconsistent use of patient name affecting meaning of report | 1 |
|  | LRG or RefSeq missing / incorrect / inconsistent | 0.5 (marks deducted once) |
| Clerical Accuracy | All essential patient identifiers present and no significant clerical errors. | 0 |
|  | Date of birth incorrect (any error) | 1 |
|  | Patient name has minor spelling error | 0.5 |
|  | Incorrect or missing patient gender | 0 |
|  | Failure to provide sample reference number | 0.5 |
|  | Report confusing or difficult to read - essential information "hidden" within the body of the text. It is recommended that critical pieces of information are highlighted in some way so that they are not overlooked. | 0 |
|  | Report confusing or difficult to read - too many pages and/or unnecessary use of images. | 0 |
|  | Pagination should be used on the report e.g. Page 1 of 1, Page 1 of 2 etc. | 0 |

***Supplementary Table 2*** *– Genotyping marking criteria applied to case 5*

| **Reported result** | **Genotyping deduction** | **Comment to laboratories** |
| --- | --- | --- |
| Both mutations detected | 0 | Correct result |
| c.2369C>T p.(Thr790Met) only detected | 0 | This sample also contained a c.2573T>G p.(Leu858Arg) mutation at approximately 0.5%. Please see scheme report for further details. |
| c.2573T>G p.(Leu858Arg) only detected and LOD for c.2369C>T p.(Thr790Met) is below 0.8% | 1 | This sample also contained a c.2369C>T p.(Thr790Met) mutation at approximately 0.8%. Please see scheme report for further details. |
| c.2573T>G p.(Leu858Arg) only detected and LOD for c.2369C>T p.(Thr790Met) is above 0.8% | 0 | This sample also contained a c.2369C>T p.(Thr790Met) mutation at approximately 0.8%. Please see scheme report for further details. |
| No mutations detected and LOD for both is below 0.5% for c.2573T>G p.(Leu858Arg) and 0.8% for c.2369C>T p.(Thr790Met) | 1 | This sample contained both a c.2573T>G p.(Leu858Arg) mutation at approximately 0.5% and a c.2369C>T p.(Thr790Met) at approximately 0.8% and the stated limits of detection of your assay indicate that both mutations should have been detected. |
| No mutations detected and LOD for both is above 0.5% for c.2573T>G p.(Leu858Arg) and 0.8% for c.2369C>T p.(Thr790Met) | 0 | This sample contained both a c.2573T>G p.(Leu858Arg) mutation at approximately 0.5% and a c.2369C>T p.(Thr790Met) at approximately 0.8%. Please see scheme report for further details. |
